# Effects of Visual Display on Joint Excursions Used to Play Virtual Dodgeball

**DOI:** 10.2196/games.6476

**Published:** 2016-09-15

**Authors:** James S Thomas, Christopher R France, Megan E Applegate, Samuel T Leitkam, Peter E Pidcoe, Stevan Walkowski

**Affiliations:** ^1^Ohio UniversitySchool of Rehabilitation and Communication SciencesAthens, OHUnited States; ^2^Ohio UniversityDepartment of PsychologyAthens, OHUnited States; ^3^Virginia Commonwealth UniversityDepartment of Physical TherapyRichmond, VAUnited States; ^4^Ohio University Heritage College of Osteopathic MedicineDepartment of Osteopathic Manipulative MedicineAthens, OHUnited States

**Keywords:** virtual reality, avatar presentation, joint kinematics

## Abstract

**Background:**

Virtual reality (VR) interventions hold great potential for rehabilitation as commercial systems are becoming more affordable and can be easily applied to both clinical and home settings.

**Objective:**

In this study, we sought to determine how differences in the VR display type can influence motor behavior, cognitive load, and participant engagement.

**Methods:**

Movement patterns of 17 healthy young adults (8 female, 9 male) were examined during games of Virtual Dodgeball presented on a three-dimensional television (3DTV) and a head-mounted display (HMD). The participant’s avatar was presented from a third-person perspective on a 3DTV and from a first-person perspective on an HMD.

**Results:**

Examination of motor behavior revealed significantly greater excursions of the knee (*P*=.003), hip (*P*<.001), spine (*P*<.001), shoulder (*P*=.001), and elbow (*P*=.026) during HMD versus 3DTV gameplay, resulting in significant differences in forward (*P*=.003) and downward (*P*<.001) displacement of the whole-body center of mass. Analyses of cognitive load and engagement revealed that relative to 3DTV, participants indicated that HMD gameplay resulted in greater satisfaction with overall performance and was less frustrating (*P*<.001). There were no significant differences noted for mental demand.

**Conclusions:**

Differences in visual display type and participant perspective influence how participants perform in Virtual Dodgeball. Because VR use within rehabilitation settings is often designed to help restore movement following orthopedic or neurologic injury, these findings provide an important caveat regarding the need to consider the potential influence of presentation format and perspective on motor behavior.

## Introduction

Virtual reality (VR) has been used to shape motion in patients with various orthopedic and neurologic impairments (eg, low back pain, cerebral vascular accident) for a number of years [1-4]. As commercial VR systems become increasingly affordable, the feasibility of applying this technology to both clinical and home settings holds great potential for clinical rehabilitation. A particular advantage of VR is that a variety of visual stimuli can be presented and manipulated in real-time to provide insights into neural control of movement and to guide or shape joint motion. However, given the variety of platforms available to present VR stimuli, it is important to better understand how differences in the VR environment can influence motor behavior, cognitive load, and participant engagement.

Levin and colleagues have examined the effects of VR environment on motor behavior in both healthy participants and stroke patients [2,4-6], and in a recent study reported no effect of display type (ie, three-dimensional [3D] image projected on a large screen vs a head-mounted display) on end-effector path straightness, shoulder and elbow joint excursions, or trunk displacements in reaches made to virtual targets from a seated posture [6]. However, differences in vertical and horizontal direction errors were noted between the display types [6]. Others have reported that displacements of the trunk and limbs during reaching tasks were altered by manipulating the viewing angle of the participant’s third-person avatar [7]. Specifically, participants reached less when the camera was oriented at 0° (ie, directly behind the avatar) than when it was oriented at angles from 45-77.5° relative to the avatar [7]. Further, the increased segment displacement observed with greater angles was accompanied by a slightly larger displacement of the whole body center of mass (COM). The results are mixed regarding the effects of VR display types on motor performance, but investigations often focused on error of the end-effector [2,4-6,8], effects of the presenting stimulus on distance judgement [9], or the influence of restricted fields of view (FOV) conditions on estimates of distances in virtual environments [9-12]. Further, these investigations have primarily focused on motor tasks that require interception with a static target and have not examined the role of virtual environments on movement strategies adopted during virtual gaming. In fact, to the authors’ knowledge no studies to date have examined how VR display types influence apportionment of joint excursions when gameplay requires significant movement of the postural joints. Finally, while some investigations have reported that the sense of actual presence in VR was weakened when the avatar was viewed from a third-person perspective [13,14], there is no evidence in the existing literature that addresses the potential effects of avatar perspective on motor behavior. However, resolving this question is particularly important for developing robust rehabilitation interventions designed to shape motor behavior.

Individuals with back pain and fear of movement due to perceived risk of harm or injury (ie, kinesiophobia) consistently avoid lumbar flexion [15-19]. Indeed, we have shown reduced lumbar flexion among participants with kinesiophobia and experimental low back pain [20], subacute low back pain [16], and chronic low back pain [18], as well as among asymptomatic individuals with kinesiophobia who have recently recovered from an episode of low back pain [17]. To address this problem, we developed a VR intervention, Virtual Dodgeball, to promote lumbar flexion in individuals with chronic low back pain and fear of movement-related injury. We recently completed a Phase I randomized controlled trial to examine feasibility and safety among individuals with chronic low back pain and high levels of pain-related fear [21]. In this initial trial, Virtual Dodgeball was played on a 3D television (3DTV) and the avatar was presented in the third-person perspective. However, because we are interested in enhancing portability of this intervention beyond the laboratory and clinic and into the home environment, this study was designed to compare movement patterns when Virtual Dodgeball is presented on a 3DTV display versus a less expensive and more portable head-mounted display (HMD). Based on existing studies, we predicted that these different display types would not affect joint excursions in full-body reaching tasks.

## Methods

### Recruitment

We recruited 17 healthy young adults (9 male, 8 female) aged 18-35. Exclusion criteria included a history of low back injury, low back pain within the last 6 months, and any orthopedic, neurological, or visual impairment that would prevent participation. This study was approved by the Institutional Review Board of Ohio University, and written informed consent was obtained at the beginning of the session. Using a within-subjects design, participation consisted of standardized reaches to static targets in the real world (RW) and a round of Virtual Dodgeball using two different visual display types (ie, 3DTV, HMD). Each round of dodgeball consisted of three levels of difficulty. Between each level, the participant had to reach to static virtual targets presented at the same locations as the corresponding reaches performed in RW. This manuscript examines the joint excursions used to intercept the launched virtual balls during Virtual Dodgeball gameplay with two different visual display types.

### Instrumentation

Movement of light-reflective marker clusters attached to the head, upper arms, forearms, hands, trunk, pelvis, thighs, shanks, and feet were tracked using a 10-camera Vicon Bonita system sampled at 100 Hz. This optoelectric-based kinematic system can track the 3D coordinates of light reflective marker clusters attached to the participant with a spatial resolution of 0.1 mm.

The time-series joint angle data were derived from the 3D segment coordinate data using an Euler angle sequence of (1) flexion-extension, (2) lateral bending, and (3) axial rotation [22] using MotionMonitor software. Joint excursions were defined as the change in joint angle from initial standing posture to posture at target contact.

### Procedures

Participants reached at a comfortable speed holding a regulation dodge ball (24 cm diameter) with both hands. They performed reaches to each of three targets located in the mid-sagittal plane. Target locations were determined for each subject based on their hip height, trunk length, and arm length. The highest target was located such that the subject could, in theory, reach the target by flexing the hips 15° with the shoulder flexed to 90° and the elbow extended. Using the same shoulder and elbow joint positions, the middle and low targets could be reached by flexing the hips 30° and 60°, respectively. Using this individualized method of determining target heights allows for comparison of movement patterns across different individuals [23-25]. We have previously demonstrated that this standardized reaching task challenges participants to produce progressively more lumbar spine flexion, and in doing so is sensitive to individual differences in movement strategies between healthy individuals and those with low back pain [15-19]. In this study, the average lumbar excursions that each participant used to reach the high, middle, and low targets was subsequently used to calculate the intended impact height location of the virtual dodgeballs (described in greater detail below).

In brief, one full game was completed in each visual display type. The order of presentation of the visual display type was randomized and counter balanced such that half the participants played Virtual Dodgeball on the 3DTV first and half played Virtual Dodgeball on the HMD first. For each participant, the impact heights of the virtual balls were identical between the visual display types. During Virtual Dodgeball, participants competed against 4 virtual opponents and the object was to block or avoid virtual balls launched randomly by each of the 4 opponents. Participants earned points and cash rewards by successfully blocking launched virtual balls using a ball that they held in their hands or by avoiding a launched ball by ducking (see Multimedia Appendix 1 for a video of gameplay).

### Virtual Environment

Vizard software (WorldViz) was used to develop the virtual environment and control all presented graphics and audio stimuli, including the opposing team’s avatars. The six degrees of freedom kinematic data from the clusters of light reflective markers placed on the participant were streamed to the game environment at 100 Hz using Vicon Tracker software to allow for near real-time presentation of the participant’s avatar (39 ms latency). The MotionMonitor software was used to control bidirectional communication with Vizard, set game parameters and target locations, and record all kinematic data during the experimental testing session.

In the 3DTV condition, a Samsung 1080p 240 Hz 3D Smart LED TV was paired with 3D shutter glasses providing an effective refresh rate of 60 Hz/eye. The participant viewed their slightly translucent avatar from a third-person perspective from a camera position 1.5 meters directly behind their avatar. The translucent avatar allowed for visibility of objects in front of the avatar. The FOV for gameplay with the 3DTV display was as follows: horizontal=50°, vertical=40°. In the HMD condition, the participant viewed their avatar from a first-person perspective that was projected using an Oculus Rift (Oculus Rift Developers Kit 2). From this perspective, the participant viewed their avatar and the environment from the position of the avatar’s eyes. The FOV for the HMD display was as follows: horizontal=100°, vertical=100°, and the refresh rate was fixed at 75 Hz/eye.

### Gameplay

**Figure 1:**
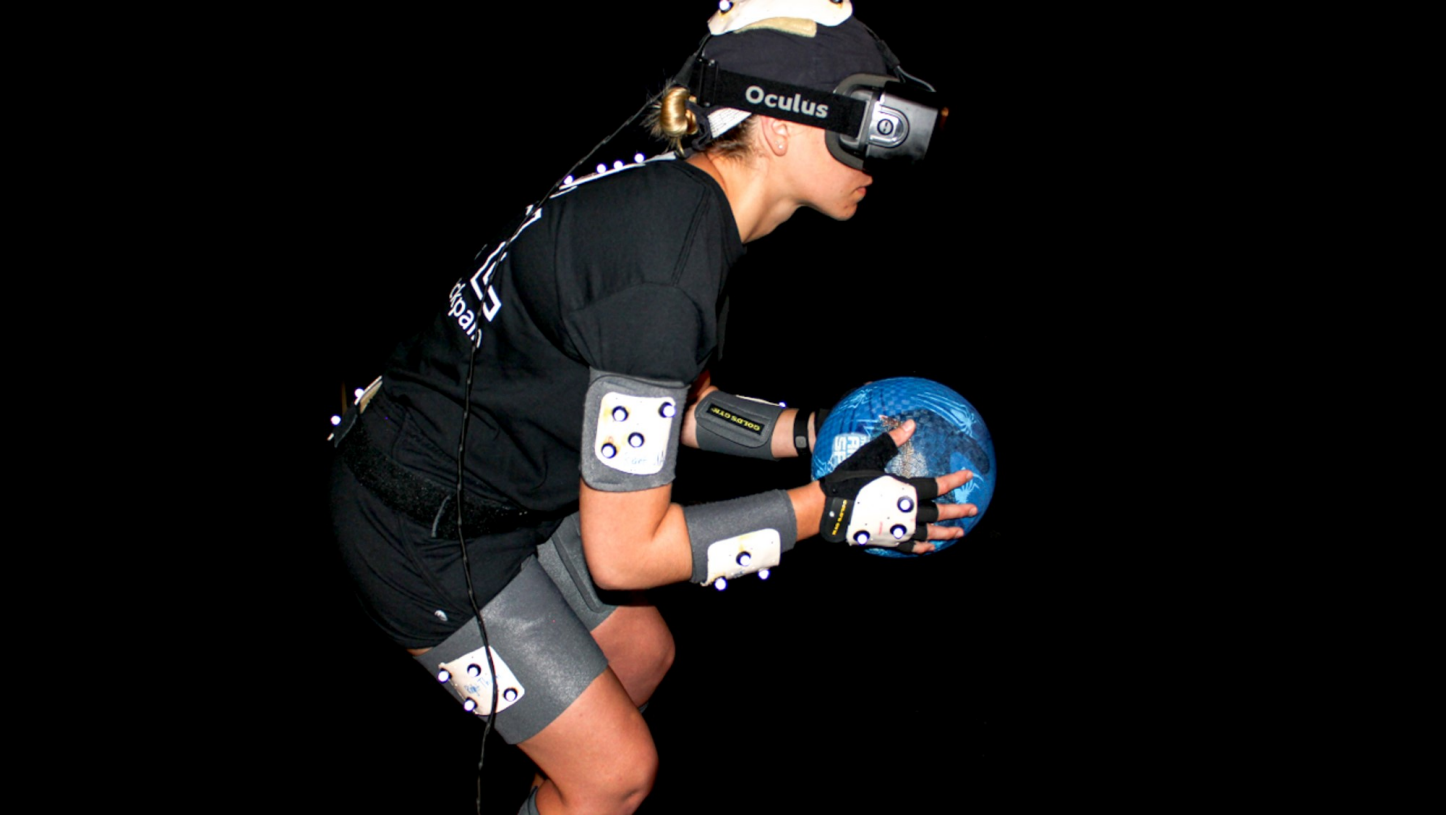
Participant instrumented and engaged in Virtual Dodgeball using the head-mounted display (HMD).

The game environment was an indoor basketball arena, with the participant positioned at the free-throw line on one side of the court and the four virtual opponents positioned on the free-throw line on the opposite side of the court. The opposing players moved 3 m fore-aft and 3 m left-right in a random order. Virtual balls were launched every 3.3 ± 0.3 seconds in a randomized order from each of the 4 virtual opponents. The opponent who was about to launch a virtual ball changed color 300 ms prior to launch to alert the participant. If the opponent turned green and the launched ball was yellow, the participant had to attempt to block the ball with the ball held in their hand (co-located with the virtual ball held by the avatar). If the opponent turned red and the launched ball was orange, the participant had to attempt to duck to avoid the ball. A large scoreboard was positioned at the opposite end of the arena (above the opponents) so that participants could track their performance and cash rewards earned. Sound effects were also incorporated, including crowd cheering, buzzers, referee whistles, and a duck quacking sound that occurred whenever an orange ball was launched. An instrumented participant engaged in virtual dodgeball with the HMD is shown in Figure 1.

**Figure 2:**
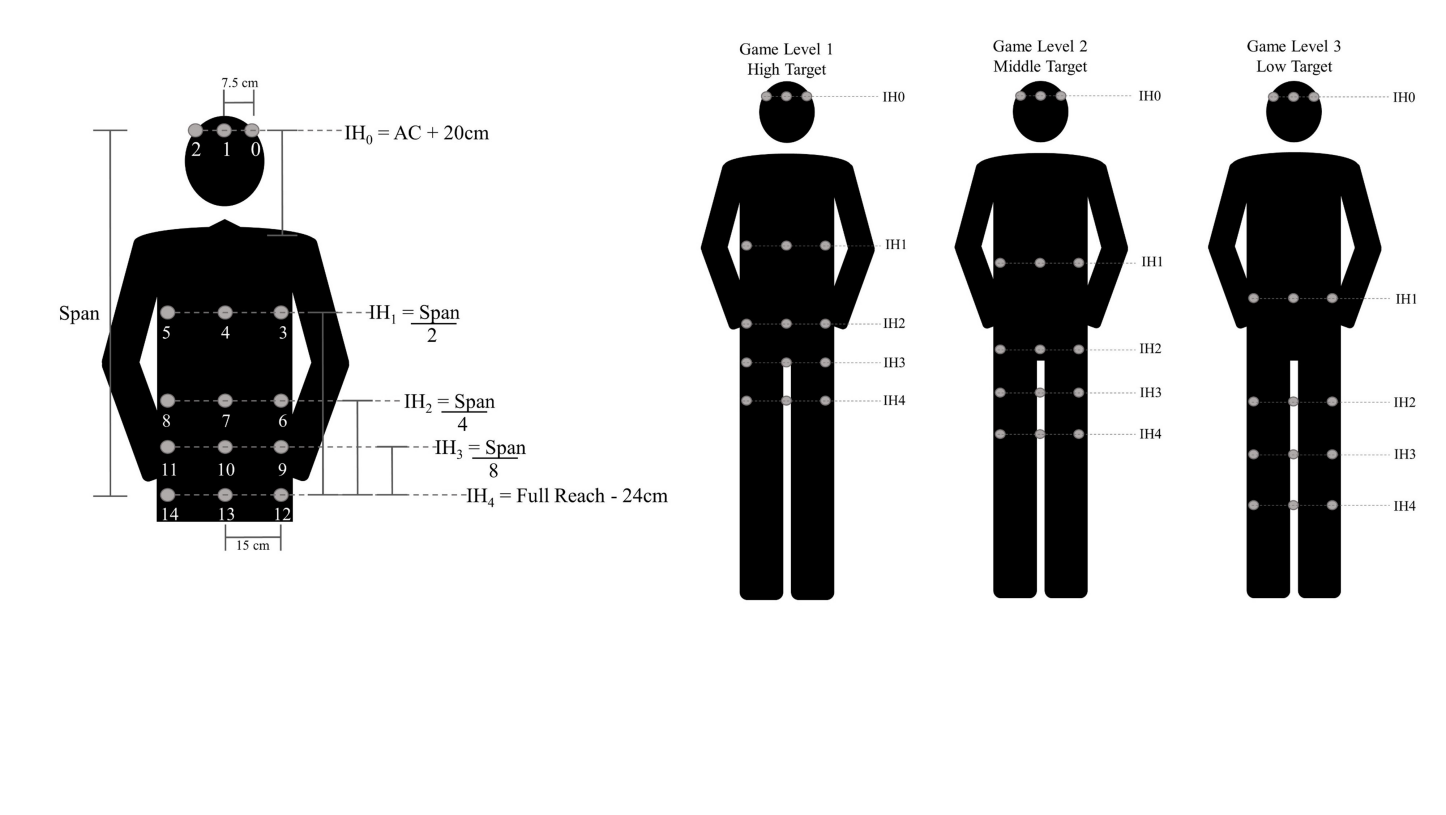
Methods for computing location of the impact heights (IH0-IH4) of the launched virtual balls for a single game level (left). The distribution of launched virtual balls across the 3 levels of gameplay is shown (right). The lowest impact height (IH4) for each gameplay level (1-3) was calculated from the lumbar spine flexion used to reach the high, middle, and low targets during the baseline standardized reaching tasks.

A round of gameplay consisted of a basic practice level to introduce the scoring metrics and three game levels, each lasting approximately 2 minutes. There were two sets of 15 launched balls within each game level. The intended impact locations of the 15 launched balls were distributed to five impact heights (IH) that were determined by the participant’s height and the amount of lumbar flexion they used during the baseline standardized reaching tasks. For example, during Level 1 of gameplay, the participant could successfully block the virtual ball launched to IH4 (ie, the lowest impact height) simply by using the identical amount of lumbar flexion used in the standardized reaching task to the high target performed at baseline, whereas during Level 3 of gameplay, the participant could successfully block the virtual ball launched to IH4 (ie, the lowest impact height) simply by using the identical amount of lumbar flexion used in the standardized reaching task to the low target performed at baseline. The five impact heights used in gameplay were scaled to impact between the height of participant’s eyes (IH0=highest impact) and approximately their shins (IH4=lowest impact) across the three levels of gameplay (see Figure 2). Three balls were launched at each IH to intersect the participant at their midline, and 20 cm left or right of the midline. It is important to note that the order of the virtual launched balls was permutated at each round of play to make the game exciting, challenging, and to some extent unpredictable. After each set, the participant was presented with a static virtual ball and instructed to reach out and touch the ball with the ball held in their hands. The location of the virtual ball was co-located to individualized target locations used during the standardized reaching task performed in real-world at pretreatment baseline such that the locations of the virtual balls during Levels 1, 2, and 3 were co-located to the real-world location of the high target, middle target, and low target, respectively.

Performance was updated in real-time and displayed on the virtual scoreboard, and the participant was awarded progressively more for each successful block or duck at each level of play (Practice Level=1¢, Level 1=2¢, Level 2=5¢, Level 3=10¢). Successful contact for each highlighted ball presented between each set resulted in a bonus 25¢ reward. Conversely, the participant lost cash rewards for each failure to block or duck. Each player started the game with a cash balance on the scoreboard such that if they failed on every launched or presented ball, their cash balance would be zero. The average gameplay session lasted approximately 15 minutes.

Following each session, the participants rated their overall efforts using the NASA Task Load Index (TLX). The NASA TLX is multidimensional assessment that rates perceived workload across to assess system performance [26] (Multimedia Appendix 2). Specifically, the NASA TLX asked the participants to provide 1 (very low) to 7 (very high) ratings of their experience along six dimensions: Mental Demand, Physical Demand, Temporal Demand, Performance, Effort, and Frustration. In the context of assessing display type on Virtual Dodgeball, this measure provides insight into differences in the perceived workload performing nearly identical tasks.

### Data Reduction and Analysis

Because the games were played with both hands in fixed locations on the ball and joint excursions were nearly identical for the left and right limbs, analyses are restricted to the right side. First, time-series position vector of the right index fingertip was smoothed using a 41-point fourth-order Savitzky-Golay filter [27]. That is, at each sample time, fourth-order polynomials were fit in the least-squares sense to the data at that point and 20 neighboring samples on each side. The polynomial coefficients were then used to determine velocity. Movement onset was determined from a backwards search from peak velocity and defined as the point where velocity was ≤5% peak velocity. Target contact was defined as the point where velocity was ≤5% peak velocity using a forward search from peak velocity. The change in joint angles (ie, ankle, knee, hip, spine, shoulder, and elbow) and displacement of whole body COM along the anterior-posterior, medio-lateral, and vertical axes was calculated from movement onset to target contact. To determine hand position at target contact, we first calculated the centroid of the hands from the x, y, and z position traces from marker clusters on the left and right hands and adjusted this to the centroid of the left and right ankle joint. We then determined the hand position at target contact for the anterior-posterior, medio-lateral, and vertical axes.

### Statistical Power and Analysis

We calculated that we needed 14 participants to determine the within-subject effects of display type with 80% power, assuming alpha=.05, and correlation between measures of .5 and an effect size of *f*=0.4 (large effect) using G*Power 3.19 [28]. The effect size was based on initial pilot testing of the effects of display types on movement strategies. The dependent measures were (1) movement time, (2) hand position at target contact (ie, anterior-posterior [AP], medial-lateral [ML], vertical), (3) joint excursions (ie, right ankle, knee, hip, spine, shoulder, and elbow), and (4) displacement of COM (ie, AP, ML, vertical). Separate 4-way mixed-model multivariate analysis of variance with sex as the between-subjects variable, and environment type (3DTV, HMD), IH (IH0-IH4), and Level (1-3) as the repeated factors were performed on the dependent measures. Posthoc analyses were performed using the method of least significant differences. Interactions were examined using a simple effects model. The NASA TLX data were analyzed used paired *t* tests with a Bonferroni correction for multiple comparisons. All statistical analyses were completed in SPSS 22.

## Results

### Movement Time

**Figure 3:**
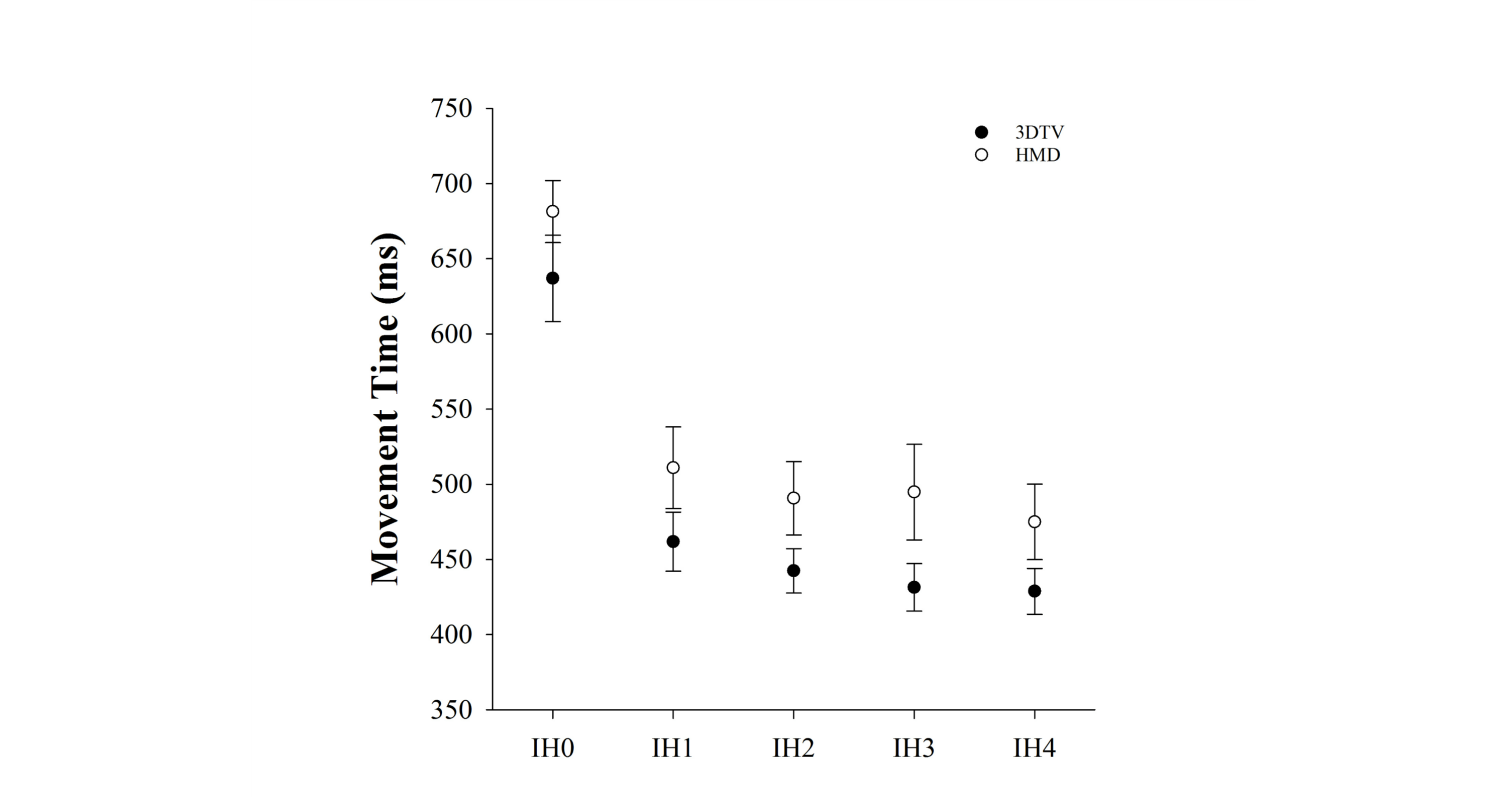
Effect of 3D television (3DTV) versus a head-mounted display (HMD) on movement time for each impact height (IH).

There was a main effect of Display Type on movement time (*F*_1,15_=6.72, *P*=.02), with participants moving more quickly in the 3DTV condition. Specifically, mean movement time for reaches made to intercept the launched virtual balls in the 3DTV condition was 480 ms (SD 140) versus 530 ms (SD 210) for the HMD condition. There was also a main effect of IH on movement time (*F*_4,12_=12.76, *P*<.001). As illustrated in Figure 3, this is driven primarily by the differences in movement times between the movements to virtual balls launched to IH0 (ie, duck condition) compared virtual balls launched to IH1-4 (ie, block conditions). Specifically, movement times for IH0 averaged 659 ms (SD 220), which was significantly longer than movement times to intercept virtual balls launched to the other targets: IH1=486 ms (SD 200) to IH4=451 ms (SD 180). But posthoc analyses revealed that there were no significant differences in movement times between any of the pairs of IH1-IH4.

### Hand Position at Ball Contact

**Figure 4:**
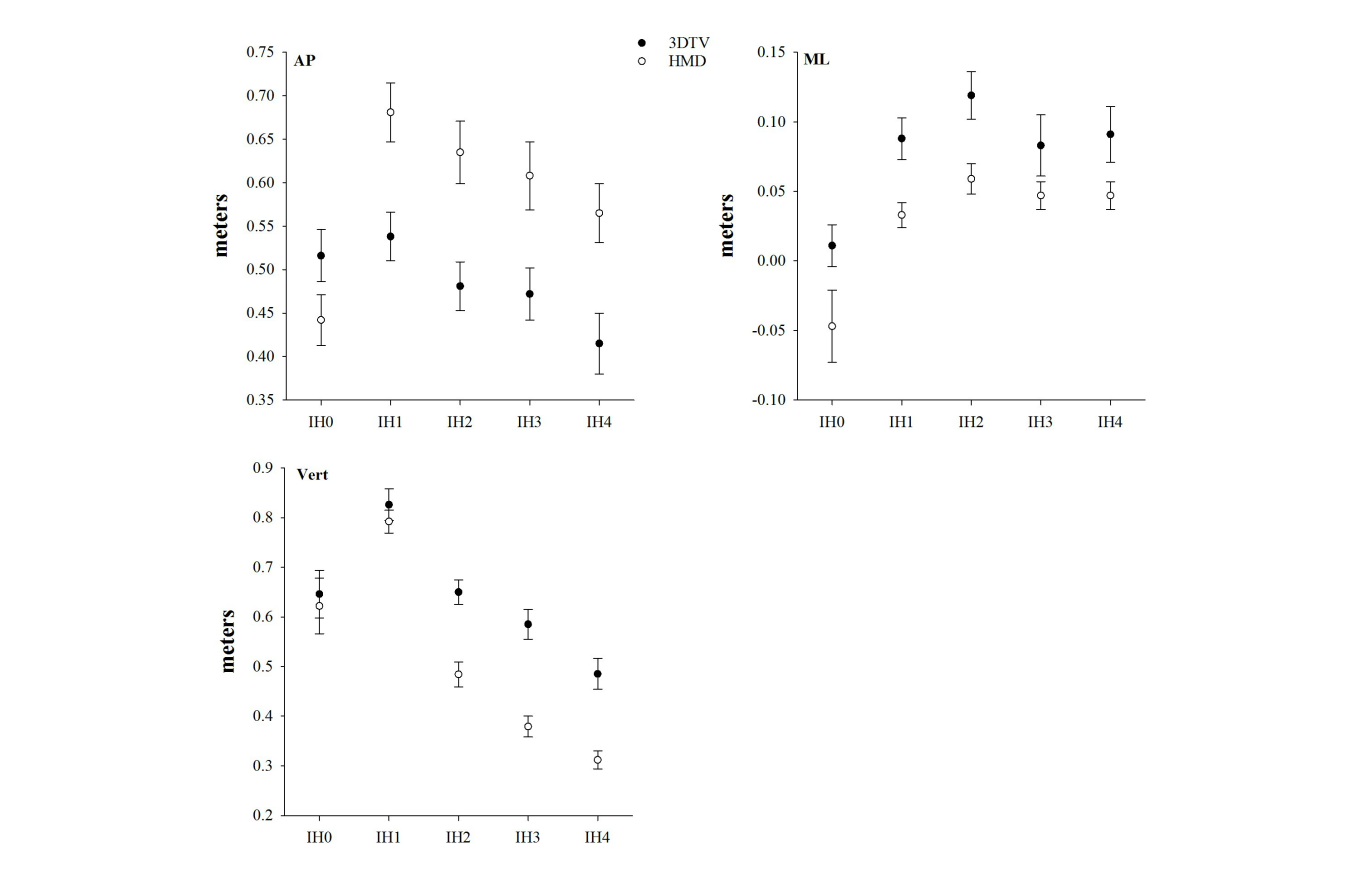
Effects of 3D television (3DTV) versus a head-mounted display (HMD) on hand position at target intercept for each impact height (IH) along the anterior-posterior (AP) axis, along the medial-lateral (ML) axis, and along the vertical axis.

As illustrated in Figure 4, there was an interaction of Display Type and IH of the launched virtual balls for the AP axis (*F*_4,12_=8.44, *P*=.002) and the vertical axis (*F*_4,12_=9.18, *P*=.01). As expected, there was also a main effect of IH on hand position at intercept with the launched virtual balls for the AP axis (*F*_1,15_=29.28, *P*<.001), the ML axis (*F*_1,15_=12.11, *P*<.001), and the vertical axis (*F*_1,15_=119.66, *P*<.001). There were also significant effects of Display Type on hand position at intercept with the launched virtual balls for the AP axis (*F*_1,15_=16.39, *P=*.001), the ML axis (*F*_1,15_=17.61, *P=*.001), and the vertical axis (*F*_1,15_=54.01, *P*<.001). Simple analyses of the effects of Display Type revealed that, compared to 3DTV, AP hand position at intercept was approximately 14 cm further forward in gameplay with HMD for IH1-4 (Multimedia Appendix 3). Vertical hand position at intercept was approximately 18 cm higher in gameplay with 3DTV compared to HMD for IH2-4 (Multimedia Appendix 3). There was no effect of Display Type for vertical hand position for IH1-2. Finally, there was a significant effect of Level, but only for the hand position along the vertical axis (*F*_1-15_=11.16, *P=*.001).

### Joint Excursions

**Figure 5:**
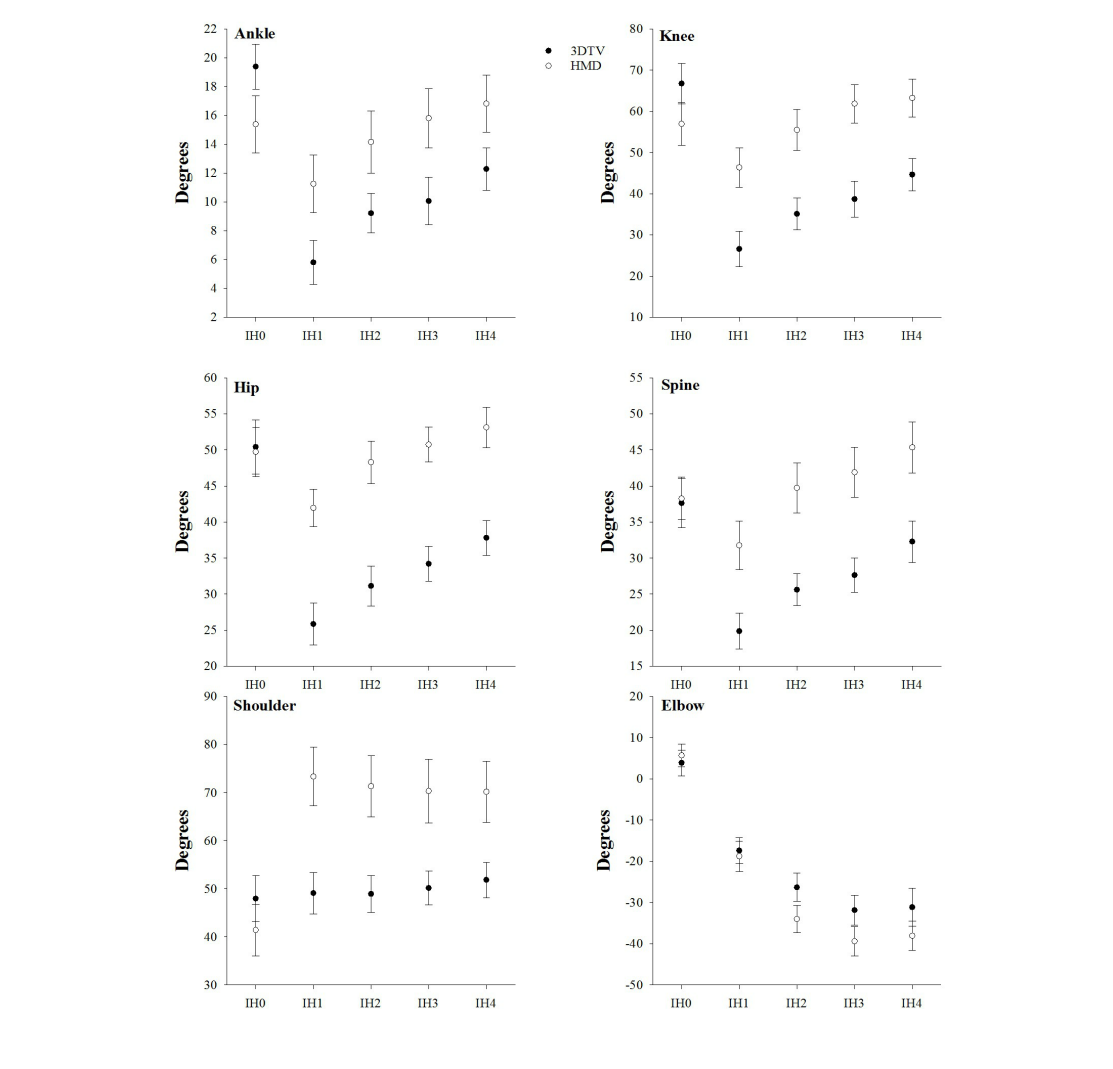
Interaction of 3D television (3DTV) versus head-mounted display (HMD) by impact height (IH) on the joint excursions of the ankle, knee, hip, spine, shoulder, and elbow.

**Figure 6:**
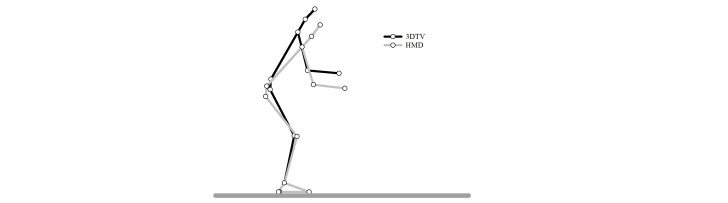
Effects of 3D television (3DTV) versus head-mounted display (HMD) on the posture adopted at target intercept.

There were significant interactions of Display Type by IH for on joint excursions of the ankle (*F*_4,12_=7.43, *P=*.003), knee (*F*_4,12_=19.00, *P*<.001), hip (*F*_4,12_=8.45, *P=*.002), spine (*F*_1,15_=5.26, *P=*.011), shoulder (*F*_4,12_=5.76, *P=*.001), and elbow (*F*_4,12_=9.95, *P*<.001). As shown in Figure 5, this interaction is driven primarily by the fact that Display Type had no effect on joint excursions for launched virtual balls to IH0 (ie, balls that required the participant to duck). For the ankle, knee, hip, spine, and shoulder, the joint excursions used to intercept the launched virtual balls to locations IH1-IH4 was significantly greater during gameplay using the HMD compared to gameplay with the 3DTV (Multimedia Appendix 3). The sum of these effects on the apportionment of joint excursions (IH1-IH4) in these full-body reaching tasks is depicted in Figure 6, which is derived from the mean join excursions across target heights, mean participant height (70”), and estimating limb segment lengths from Winter [29].

### Displacement of Center of Mass

**Figure 7:**
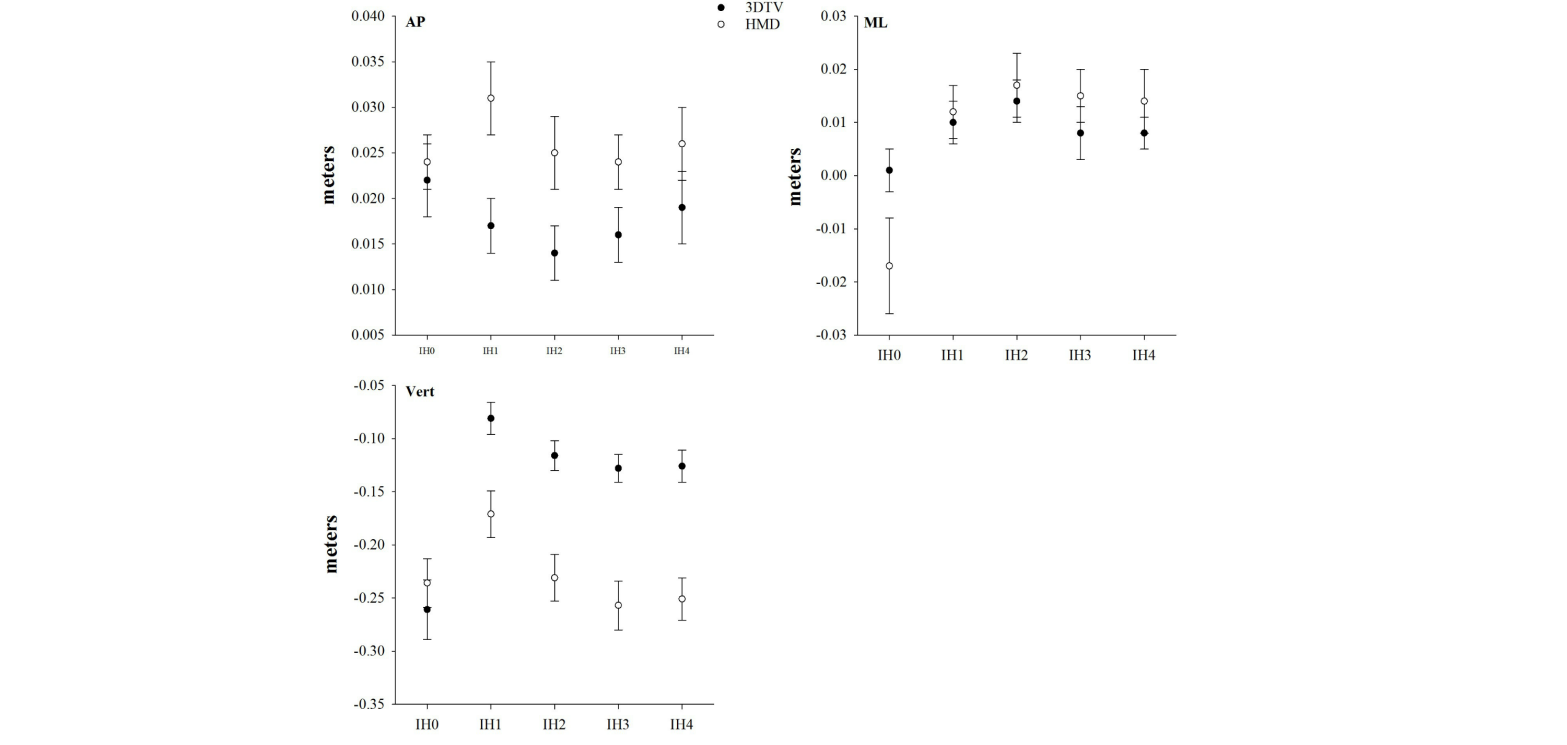
Effects of 3D television (3DTV) versus a head-mounted display (HMD) on displacement of whole-body center-of-mass (COM) for each impact height (IH) along the anterior-posterior (AP) axis, along the medial-lateral (ML) axis, and along the vertical axis.

There was a significant interaction of Display Type and IH on displacement of COM along the AP axis (*F*_4,12_=5.63, *P=*.001), ML axis (*F*_4,12_=6.03, *P=*.001), and vertical axis (*F*_4,12_=9.95, *P=*.001). As shown in Figure 7, with the exception of IH0, COM displacement along the AP and vertical axes was greater in HMD compared to 3DTV for launched virtual balls launched to IH1-4 (Multimedia Appendix 3). Conversely, the only significant effects of Display Type on COM displacement along the ML axis was for launched virtual balls to IH0. Further, the differences along the ML axis were rather small (ie, ˂2 cm). There was also a main effect of Display Type on displacement of COM along the AP axis (*F*_1,15_=12.64, *P=*.003) and vertical axis (*F*_1,15_=41.82, *P*<.001). On average, participants had an 8 cm (SD 0.003) larger forward displacement of the COM along AP axis and an 8.6 cm (SD 0.017) larger downward displacement along the vertical axis during gameplay with the HMD compared to the 3DTV.

### Task Load Index

**Figure 8:**
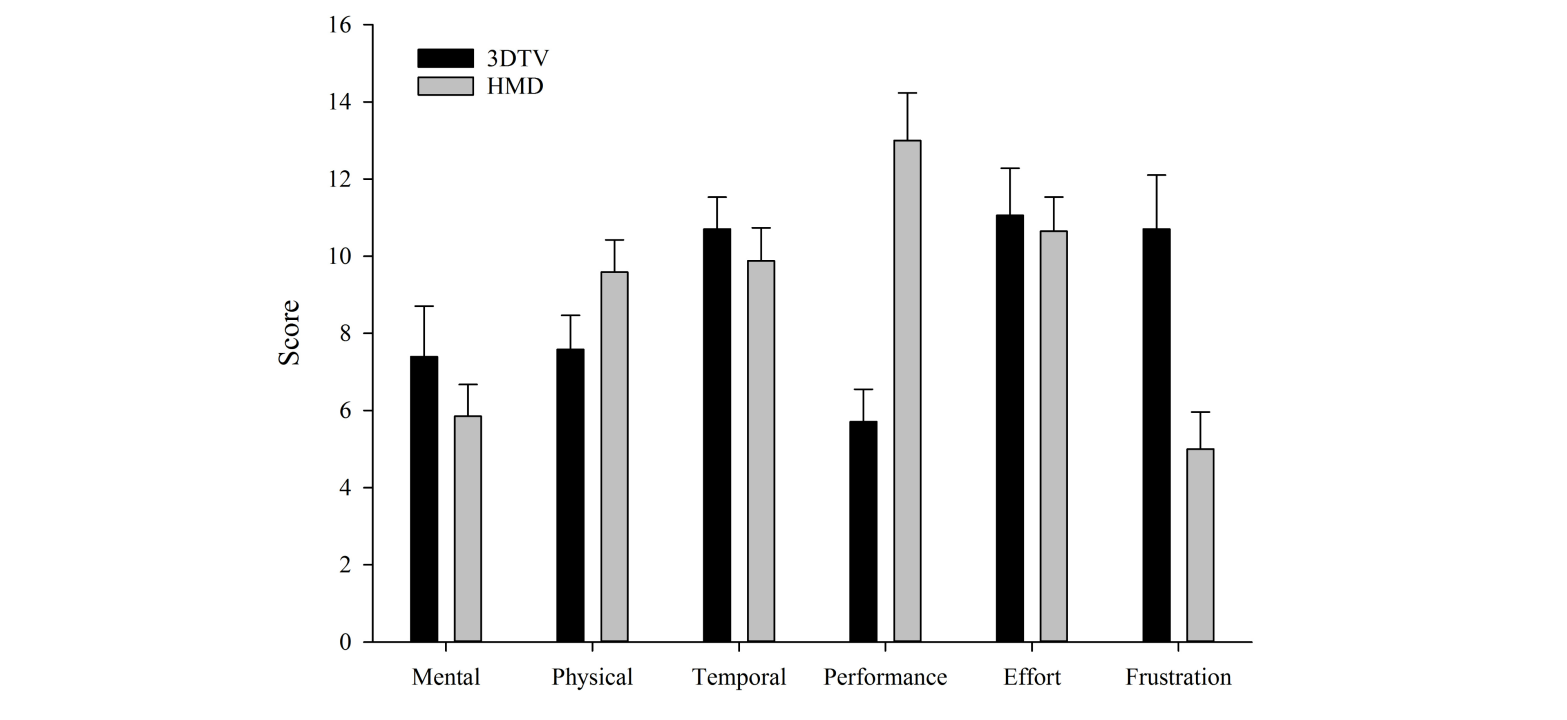
Effects of 3D television (3DTV) versus head-mounted display (HMD) on NASA Task Load Index(TLX) scores.

Analysis of the Task Load Index data (Figure 8) revealed that, relative to the 3DTV, participants indicated that HMD gameplay resulted in greater satisfaction with overall performance (*P*<.001) and was less frustrating (*P*=.001). There were no significant differences noted for physical demand, mental demand, temporal demand (ie, perceived time pressure), or overall effort required. Examination of the effects of display type on success rate during gameplay revealed that participants had a success rate of 38.8% (SD 2.0) for gameplay with the 3DTV compared to 71.2% (SD 2.6) for the HMD (*F*_1,15_=142. 4, *P<*.001).

## Discussion

### Principal Results

The primary goal of this study was to determine the effects of display type on the joint excursions used while playing Virtual Dodgeball. Figure 4 best captures how display type influences motor behavior during gameplay. When playing Virtual Dodgeball with the HMD, participants had larger excursions of the postural joints compared to gameplay on the 3DTV. This appears to reflect an overall shift in how participants respond to the launched virtual balls when using the HMD such that they intercept the virtual balls further in front of their body and from a lower position.

For virtual balls launched to IH1-4, hand position at target intercept contact was about 14 cm forward (AP axis) and 18 cm lower (vertical axis) in gameplay with HMD compared to 3DTV (Figure 2). It has been suggested that distance is underestimated in virtual reality space due, in part, to a restricted FOV [9]. However, the FOV for the 3DTV (horizontal=50°, vertical=40°) is considerably less than the FOV for the HMD (horizontal=100°, vertical=100°). If these results were driven by FOV, then one would expect target intercept with HMD to be less than 3DTV; however, that is clearly not what we found. Thus, FOV does not provide a plausible explanation for the differences in hand position at target intercept between the visual displays. This finding really indicates a difference in strategy, as it is a robust finding across the various IHs of the virtual launched balls. The exception is, of course, for the virtual balls launched to IH0 (ie, required the participant to duck). There was no difference in movement strategies as a function of display type for this portion of Virtual Dodgeball.

While differences in FOV do not provide an explanation for the differences in hand position or joint excursions, perhaps COM displacement can explain the changes in strategy between display types. The vertical displacement for the COM was greater in HMD compared to 3DTV across IH1-4. From an energetics perspective, lowering the height of the COM could result in a fundamentally more stable system. However, the forward displacement of the whole-body COM was also greater in the HMD condition compared to 3DTV. Thus, from the same energetics perspective, the greater forward displacement would not lead to a more stable system. It has been shown that COM displacement is changed in standing reaching tasks performed in virtual reality environments when the viewing angle of the participant’s avatar is altered [7]. The change in viewing angle of the avatar could have similar effects on the perception of the task and the evaluation of a participant’s location in the virtual environment [13]. Further, while Levin and colleagues found no differences in joint excursions of the trunk, shoulder, and elbow in reaching tasks performed in two visual display types (ie, 3DTV versus HMD) [2], they used a seated reaching task that did not present the same challenges to stability as intercepting dynamic targets performed from a standing position. It does not appear that conservation of displacement of COM is a significant factor in the difference in movement strategies observed in Virtual Dodgeball played with two display types.

It is possible that differences in joint excursions between the visual displays could be driven, in part, by differences in movement time to intercept the virtual launched balls. We have shown that joint excursions of the ankle, knee, and hip increase as movement time to target is reduced by half (ie, when participants move twice as fast to the target) [23,25]. However, we found that movement times were shorter during Virtual Dodgeball played with the 3DTV compared to the HMD (ie, faster movement speeds). Thus, one could expect that joint excursions would be greater in 3DTV compared to HMD. Because we observed just the opposite, movement speed does not appear to explain the differences in joint excursions observed between the visual displays.

Another potential contributor to the differences in observed movement strategies is the difference in refresh rates for the two display types. However, the kinematic input streams to the avatars in both display types is 100 Hz with the 3DTV, paired with the shutter goggles, having an effective update rate of 60 Hz/eye and the HMD having an update rate of 75 Hz. Thus the difference in refresh rates results in an absolute time difference of about 3.3 ms. Further, as both display types use an LED display, there should be no differences in persistence of the displayed images. Finally, according to Ware, the processing time for humans is approximately 166 ms and visual lags effect performance at about 200 ms [30]. Thus, it is highly unlikely that difference in refresh rates was a driving factor of the difference in joint excursions reported.

The difference in movement strategies observed between the two display types could be due to the presentation of the avatar. Some investigations have reported that the sense of actual presence in VR was weakened when the avatar was viewed from a third-person perspective [13,14]. Although differences in sense of presence could influence motor behavior, a recent study found no differences in temporal or spatial performance in a task that required participants to search and walk toward targets in the VR environment [31]. However, Ustinova and colleagues reported that trunk and peripheral joint excursions changed as a function of viewing angle of the avatar in a VR presented on a 3DTV [7], but in that study the avatar was always presented in the third-person perspective. As such, it was not a specific comparison between first- and third-person perspectives as in the current study. In fact, we are unaware of any studies that have examined the effects of avatar perspective on joint excursions. As noted in the methods, Virtual Dodgeball gameplay in 3DTV presented the participant’s avatar in the third-person perspective whereas a first-person perspective of the avatar was used in HMD. The visual transformation that must occur while viewing one’s avatar, which in the virtual world was located 1.5 meters in front of the participant, can have significant effects on movement control. However, it is possible that display type was the driving factor of the observed differences in joint excursions and not avatar perspective.

Finally, the results of this study provide further support for Virtual Dodgeball as an effective strategy to promote lumbar flexion. Importantly, the current findings also indicate that the clinical utility of Virtual Dodgeball may be enhanced with an HMD because it elicits more lumbar spine flexion, greater participant satisfaction with overall performance, and less frustration.

### Limitations

A limitation of this study is that it cannot assign differences in motor performance in these tasks simply to avatar perspective. The differences could also be due to the use of a 3DTV versus the HMD, or to differences in display of the avatar (ie, first-person versus third-person perspective). Accordingly, future studies are needed to carefully isolate the effects of perspective and display type.

### Conclusions

The results of this study demonstrate that visual display type influences motor behavior in Virtual Dodgeball. These data are important for the development of virtual reality assessment and treatment tools that are becoming increasingly practical for home and clinic use. Because a primary goal of virtual reality within rehabilitation is often to restore movement following orthopedic or neurologic injury, it is important to understand how presentation of the avatar or, by extension, camera position will affect motor behavior regardless of the display through which it is presented (ie, 3DTV or HMD). Use of home devices such as the Kinect sensor to track and presents an avatar in a third-person perspective may result in very different motor behavior when compared to the same tasks being presented from a first-person perspective.

## Multimedia Appendix 1

Video files.

## Multimedia Appendix 2

The NASA-TLX survey instrument.

## Multimedia Appendix 3

Supplementary tables.

## Abbreviations

3DTV: three-dimensional television
AP: anterior posterior
COM: center of mass
FOV: field of view
HMD: head-mounted display
ML: medial lateral
VR: virtual reality
TLX: task load index

## References

[1] Fung J, Richards CL, Malouin F, McFadyen BJ, Lamontagne A (2006). A treadmill and motion coupled virtual reality system for gait training post-stroke. Cyberpsychol Behav.

[2] Magdalon EC, Michaelsen SM, Quevedo AA, Levin MF (2011). Comparison of grasping movements made by healthy subjects in a 3-dimensional immersive virtual versus physical environment. Acta Psychol (Amst).

[3] Levin M, Magdalon E, Michaelsen S, Quevedo A (2008). Comparison of Reaching and Grasping Kinematics in Patients with Hemiparesis and in Healthy Controls in Virtual and Physical Environments.

[4] Subramanian S, Knaut LA, Beaudoin C, McFadyen B, Feldman A, Levin A (2007). Virtual reality environments for post-stroke arm rehabilitation. J Neuroengineering Rehabil.

[5] Subramanian S, Knaut L, Beaudoin C, Levin M (2007). Enhanced feedback during training in virtual versus real world environments. Virtual Rehabilitation.

[6] Subramanian SM (2011). Viewing medium affects arm motor performance in 3D virtual environments. J Neuroeng Rehabil.

[7] Ustinova KI, Perkins J, Szostakowski L, Tamkei LS, Leonard WA (2010). Effect of viewing angle on arm reaching while standing in a virtual environment: potential for virtual rehabilitation. Acta Psychol (Amst).

[8] Knaut LA, Subramanian SK, McFadyen BJ, Bourbonnais D, Levin MF (2009). Kinematics of pointing movements made in a virtual versus a physical 3-dimensional environment in healthy and stroke subjects. Arch Phys Med Rehabil.

[9] Willemsen P, Colton M, Creem-Regehr S, Thompson W (2009). The Effects of Head-Mounted Display Mechanical Properties and Field of View on Distance Judgments in Virtual Environments. Acm Transactions on Applied Perception.

[10] Creem-Regehr SH, Willemsen P, Gooch AA, Thompson WB (2005). The influence of restricted viewing conditions on egocentric distance perception: implications for real and virtual indoor environments. Perception.

[11] Thompson W, Willemsen P, Gooch A, Creem-Regehr S, Loomis J, Beall A (2004). Does the quality of the computer graphics matter when judging distances in visually immersive environments?. Presence-Teleoperators and Virtual Environments.

[12] Willemsen P, Gooch A, Thompson W, Creem-Regehr S (2008). Effects of Stereo Viewing Conditions on Distance Perception in Virtual Environments. Presence: Teleoperators and Virtual Environments.

[13] Lenggenhager B, Tadi T, Metzinger T, Blanke O (2007). Video ergo sum: manipulating bodily self-consciousness. Science.

[14] Slater M, Spanlang B, Sanchez-Vives MV, Blanke O (2010). First person experience of body transfer in virtual reality. PLoS One.

[15] Thomas JS, France CR (2007). Pain-related fear is associated with avoidance of spinal motion during recovery from low back pain. Spine (Phila Pa 1976).

[16] Thomas JS, France CR (2008). The relationship between pain-related fear and lumbar flexion during natural recovery from low back pain. Eur Spine J.

[17] Thomas JS, France CR, Lavender SA, Johnson MR (2008). Effects of fear of movement on spine velocity and acceleration after recovery from low back pain. Spine (Phila Pa 1976).

[18] Thomas JS, France CR, Sha D, Vander WN, Moenter S, Swank K (2007). The effect of chronic low back pain on trunk muscle activations in target reaching movements with various loads. Spine (Phila Pa 1976).

[19] Thomas JS, France CR, Sha D, Wiele NV (2008). The influence of pain-related fear on peak muscle activity and force generation during maximal isometric trunk exertions. Spine (Phila Pa 1976).

[20] Trost Z, France CR, Sullivan MJ, Thomas JS (2012). Pain-related fear predicts reduced spinal motion following experimental back injury. Pain.

[21] Thomas JS, France CR, Applegate ME, Leitkam ST, Walkowski S (2016). Feasibility and Safety of a Virtual Reality Dodgeball Intervention for Chronic Low Back Pain: A Randomized Clinical Trial. J Pain.

[22] McGill SM, Cholewicki J, Peach JP (1997). Methodological considerations for using inductive sensors (3SPACE ISOTRAK) to monitor 3-D orthopaedic joint motion. Clin Biomech (Bristol, Avon).

[23] Thomas JS, Corcos DM, Hasan Z (2003). Effect of movement speed on limb segment motions for reaching from a standing position. Exp Brain Res.

[24] Thomas JS, Corcos DM, Hasan Z (2005). Kinematic and kinetic constraints on arm, trunk, and leg segments in target-reaching movements. J Neurophysiol.

[25] Thomas JS, Gibson GE (2007). Coordination and timing of spine and hip joints during full body reaching tasks. Hum Mov Sci.

[26] Hart S (2006). NASA-Task Load Index (NASA-TLX); 20 Years Later.

[27] Press WH, Teukolsky SA, Vetterling WT, Flannery BP (1993). Numerical recipes in FORTRAN: the art of scientific computing.

[28] Faul F, Erdfelder E, Lang A, Buchner A (2007). G*Power 3: a flexible statistical power analysis program for the social, behavioral, and biomedical sciences. Behav Res Methods.

[29] Winter DA (2009). Biomechanics and motor control of human movement.

[30] Ware CR (1994). Target Acquisition in Fish Tank VR - the Effects of Lag and Frame Rate.

[31] Török A, Nguyen TP, Kolozsvári O, Buchanan RJ, Nadasdy Z (2014). Reference frames in virtual spatial navigation are viewpoint dependent. Front Hum Neurosci.

