# Supplementary material for: Effects of Visual Display on Joint Excursions Used to Play Virtual Dodgeball
Source: JMIR Serious Games. 2016 Sep 15;4(2):e16. doi: 10.2196/games.6476 (PMC5043121; doi:10.2196/games.6476)
Supplement: Supplementary file 1 [file games_v4i2e16_app1.pdf]

Multimedia Appendix 1

The following clip shows a graduate student playing Virtual Dodgeball with the 3DTV: https://www.youtube.com/watch?v=1QV8sJJQQ8Q

The following clip shows the same graduate student playing Virtual Dodgeball with the HMD: https://www.youtube.com/watch?v=5q7ppnOtMb8
