# Supplementary figures and images for: Effects of Visual Display on Joint Excursions Used to Play Virtual Dodgeball
Source: JMIR Serious Games. 2016 Sep 15;4(2):e16. doi: 10.2196/games.6476 (PMC5043121; doi:10.2196/games.6476)

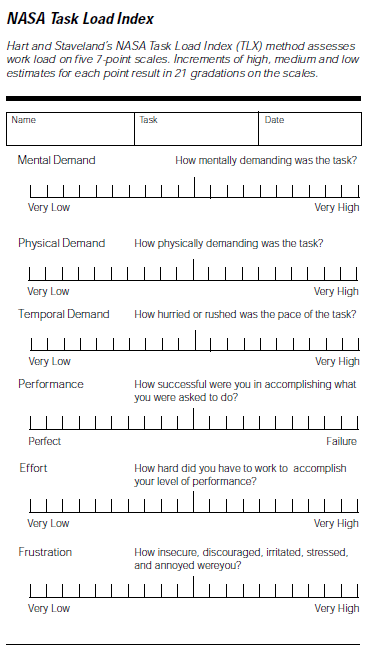

Supplement: Supplementary file 2 [file games_v4i2e16_app2.png]
