# Supplementary material for: Effects of Visual Display on Joint Excursions Used to Play Virtual Dodgeball
Source: JMIR Serious Games. 2016 Sep 15;4(2):e16. doi: 10.2196/games.6476 (PMC5043121; doi:10.2196/games.6476)
Supplement: Supplementary file 3 [file games_v4i2e16_app3.pdf]

Multimedia Appendix 2

Table 1. Simple effects of Display Type at each IH for hand position at target intercept.

Hand Position	IH1	IH2	IH3	IH4
AP	*P*<.001	*P*=.001	*P*=.001	*P*=.001
ML	*P*=.002	*P*=.012		*P*=.013
Vert		*P*<.001	*P*<.001	*P*<.001

Table 2. Simple effects of Display Type at each IH for joint excursions.

Joint	IH1	IH2	IH3	IH4
Ankle	*P*=.02	*P*=.025	*P*=.006	*P*=.006
Knee	*P*=.002	*P*=.002	*P*<.001	*P*=.001
Hip	*P*<.001	*P*<.001	*P*<.001	*P*<.001
Spine	*P*<.001	*P*<.001	*P*<.001	*P*=.001
Shoulder	*P*<.001	*P*<.001	*P*=.001	*P*=.002
Elbow		*P*<.001	*P*=.022	

Table 3. Simple effects of Display Type at each IH for change in COM.

COM	IH1	IH2	IH3	IH4
AP	*P*<.001	*P*=.004	*P*=.008	*P*=.04
ML				
Vert	*P*=.001	*P*<.001	*P*<.001	*P*<.001
